# Exosomes and non-coding RNAs in the regulation of neuroinflammation after ischemic stroke: mechanisms and therapeutic perspectives

**DOI:** 10.3389/fimmu.2025.1601843

**Published:** 2025-09-04

**Authors:** Dekai Wei, Fujun Li, Chunhui Guo, Jibing Chen, Yanqiu You

**Affiliations:** ^1^ Graduate School, Guangxi University of Chinese Medicine, Nanning, China; ^2^ Ruikang Hospital affiliated to Guangxi University of Chinese Medicine, Nanning, China; ^3^ Department of Anesthesiology, Sichuan Clinical Research Center for Cancer, Sichuan Cancer Hospital & Institute, Sichuan Cancer Center, Affiliated Hospital of University of Electronic Science and Technology of China, Chengdu, China

**Keywords:** ischemic stroke, ncRNAs, exosome, neuroinflammation, ischemia/reperfusion (I/R) injury

## Abstract

Ischemic stroke, one of the cerebrovascular diseases with the highest global disability and mortality rates, is characterized by secondary neuroinflammatory injury during its pathological progression, which remains a major challenge in clinical management. Although reperfusion therapies, including intravenous thrombolysis (IVT) and endovascular mechanical thrombectomy (EVT), have significantly improved acute-phase blood flow restoration, the neuroinflammatory cascade triggered post-reperfusion exacerbates neuronal damage. Key mechanisms include microglial overactivation and blood-brain barrier (BBB) disruption, ultimately leading to poor neurological outcomes. Recent studies have increasingly revealed the pivotal roles of exosomes and non-coding RNAs (ncRNAs) in post-ischemic stroke pathology. Specifically, exosomes, as natural nanocarriers, demonstrate targeted regulation of immune-inflammatory cascades in cerebral ischemia/reperfusion injury due to their low immunogenicity and efficient delivery capacity; complementarily, ncRNAs participate in pathophysiological processes including apoptosis, angiogenesis, inflammatory responses, and hypoxic stress through epigenetic regulatory mechanisms. This review systematically deciphers the regulatory networks of exosomes and ncRNAs in post-stroke pathological progression and neural repair, with particular focus on their molecular mechanisms in modulating specific inflammatory components. Building on current advances, we emphasize that while affirming the clinical value of reperfusion therapy, it is imperative to integrate evidence-based secondary prevention systems to address stroke management challenges. Notably, exosome-derived ncRNAs have emerged as promising diagnostic/therapeutic candidates: they not only precisely regulate inflammation-related pathways but also provide a novel strategy for developing targeted delivery systems. With deepening mechanistic understanding, exosome-based therapies are expected to revolutionize therapeutic paradigms for neuroinflammatory disorders, paving new avenues for precise intervention and functional recovery in stroke patients.

## 1 Introduction

Stroke, the second most common fatal disease worldwide and the leading cause of permanent disability in adults, encompasses hemorrhagic stroke (HS) and ischemic stroke (IS). IS, which accounts for 87% of cases, has its core pathological mechanism involving acute arterial occlusion-induced cerebral ischemia and hypoxia (1–4). Current clinical management of acute IS emphasizes time window-dependent vascular recanalization, predominantly relying on IVT, EVT, or their combined application. Neurons are highly sensitive to oxygen-glucose deprivation; therefore, timely restoration of blood perfusion is critical to mitigate irreversible damage. Although thrombolytic agents such as alteplase (requiring administration within 4.5 hours post-onset) and modified tenecteplase can dissolve thrombi, delayed symptom recognition and prolonged medical transfers prevent most patients from benefiting from thrombolytic therapy (5–7). In contrast, EVT extends the therapeutic window to 24 hours through physical thrombus removal, further improving outcomes in patients with large vessel occlusion (8–11). Nevertheless, fewer than 3% of global stroke patients receive EVT, with a 460-fold disparity in technical accessibility between nations, underscoring the critical imbalance in healthcare resource allocation (12).

Notably, even with successful reperfusion, nearly half of patients experience poor prognosis due to reperfusion injury, while current antiplatelet therapies merely prevent stroke recurrence without repairing existing neural damage (13–16). Three major bottlenecks constrain contemporary IS treatment: (1) Strict time windows exclude most patients from intervention opportunities; (2) The BBB blocks delivery of 95% of neurorestorative drugs; (3) Effective interventions for reperfusion injury remain elusive (17, 18). These challenges demand innovative therapeutic strategies. Exosomes, with their unique BBB-penetrating capacity, multi-target regulatory properties, and nanoscale carrier advantages, provide a groundbreaking solution to simultaneously overcome drug delivery barriers, promote neural repair, and modulate inflammatory injury.

Extracellular vesicles (EVs) constitute a heterogeneous population of membrane-bound particles released by both prokaryotic and eukaryotic cells (19). EV classification depends on biogenesis pathways: exosomes (40–100 nm) formed through multivesicular body exocytosis, ectosomes (50-1,000 nm) generated via plasma membrane budding, and apoptotic bodies (800-5,000 nm) shed during programmed cell death (20, 21). As the most therapeutically promising EV subset, exosomes inherit lipid bilayers from parental cell membranes and carry diverse functional biomolecules (proteins, lipids, nucleic acids), executing essential intercellular signaling missions (22, 23). In cerebral ischemia pathology, exosomal neuroprotection stems from bioactive cargo-microenvironment interactions. Protein and nucleic acid components directly activate neurogenesis pathways, suppress microglial hyperactivation, and promote angiogenesis with BBB and white matter stabilization. Additionally, inflammatory mediators (e.g., TNF-α, IL-6) from ischemic lesions enhance exosomal chemotaxis via receptor-mediated trans-barrier migration, increasing their accumulation at injury sites to coordinate reparative and immunomodulatory responses (24–28). Compared to synthetic nanocarriers, exosomes exhibit superior targeting precision, biocompatibility, and low immunogenicity (29, 30). These intrinsic advantages position them as ideal vectors to overcome neurorestorative drug delivery barriers, offering novel technological pathways for personalized stroke therapeutics.

ncRNAs represent a class of regulatory RNA molecules lacking protein-coding potential, primarily comprising microRNAs (miRNAs), long non-coding RNAs (lncRNAs), and circular RNAs (circRNAs) (31). Accumulating evidence demonstrates that ncRNAs directly or indirectly participate in diverse pathophysiological processes of IS, critically regulating apoptosis, vascular remodeling, inflammatory cascades, and hypoxic stress responses (32–36). The interactions among ncRNAs reveal sophisticated molecular crosstalk. Specifically, circRNAs and lncRNAs function as molecular sponges to sequester miRNAs, thereby attenuating miRNA-mRNA binding capacity and reversing miRNA-mediated negative regulation of target mRNAs. This post-transcriptional regulatory paradigm, termed the competing endogenous RNA (ceRNA) mechanism, establishes multi-layered gene expression control networks (37–39). For instance, the circRNA-miRNA-mRNA axis orchestrates fundamental cellular processes such as immune homeostasis maintenance, cell cycle progression, and stress adaptation through miRNA activity modulation (40, 41). Such networked regulatory mechanisms open new avenues for deciphering the molecular pathogenesis of IS and developing precision-targeted therapeutic strategies.

To systematically elucidate the roles of exosomes and ncRNAs in post-ischemic stroke neuroinflammation, we searched the PubMed database from January 2022 to March 2025 using the following query: “exosomes” OR “ncRNAs” OR “circRNA” OR “lncRNA” OR “miRNA” AND “stroke” AND “neuroinflammation” OR “microglia” OR “inflammation”. A three-step screening process was performed:

1. Initial search: 1,221 records identified
2. Title/abstract screening: 534 potentially eligible studies retained
3. Full-text assessment: 46 studies ultimately included based on the criteria below

### 1.1 Inclusion and exclusion criteria

#### 1.1.1 Inclusion criteria

1. Disease models: Rodent ischemic stroke models or OGD/R/LPS-induced neuroinflammatory cell models
2. Study subjects: Exosomes or ncRNAs (circRNA, lncRNA, miRNA)
3. Study type: Original mechanistic/interventional studies (excluding non-empirical literature)
4. Publication requirement: Full-text articles in English
5. Data completeness: For animal studies, explicit reporting of both neuroinflammatory markers (e.g., TNF-α, IL-1β) and infarct volume data; for cell studies, explicit reporting of neuroinflammatory markers (e.g., TNF-α, IL-1β, IL-6) and cell viability/cytotoxicity data (e.g., CCK-8, LDH release, apoptosis rate)
6. Literature prioritization: For overlapping studies, the most recent or data-complete version was retained

#### 1.1.2 Exclusion criteria

1. Methodological flaws: Absence of control groups, undisclosed animal strains or cell sources
2. Irrelevant publication types: Reviews, conference abstracts, commentaries, or studies with inaccessible full texts
3. Non-ischemic stroke models: Intracerebral hemorrhage, Alzheimer’s disease, etc.

## 2 Pathogenesis of IS and neuroinflammatory mechanisms

### 2.1 Pathophysiological basis of IS

IS occurs when cerebral blood vessels supplying the brain become obstructed. Occlusion caused by locally formed clots within cerebral vessels is termed cerebral thrombosis, which is closely associated with lipid deposits in atherosclerotic arteries. Alternatively, clots originating from extracranial sites, termed cerebral embolism, may travel through the bloodstream to cerebral vessels, with atrial fibrillation being the primary etiology (42). Due to the structural characteristics of cerebral vasculature and its hierarchical perfusion network, vascular occlusion typically manifests as focal neurological deficits confined to specific brain regions supplied by the affected vessel. The central core region experiences near-complete cessation of cerebral blood flow (CBF), resulting in rapid cellular necrosis within minutes. Surrounding this core lies a transitional zone termed the “penumbra,” where reduced perfusion permits temporary cellular survival. This salvageable tissue constitutes the primary target for neuroprotective interventions (43). Following temporary CBF deprivation in the penumbra, reperfusion restores oxygen and nutrient supply but paradoxically induces significant secondary tissue damage through ischemia-reperfusion (I/R) injury (42, 44). This dual-phase injury mechanism is not unique to cerebral ischemia but has been well-documented in other organ systems, including myocardial infarction and acute kidney injury (45–48).

### 2.2 Pathological characteristics of neuroinflammation and its multidimensional regulatory networks

**Figure 1:**
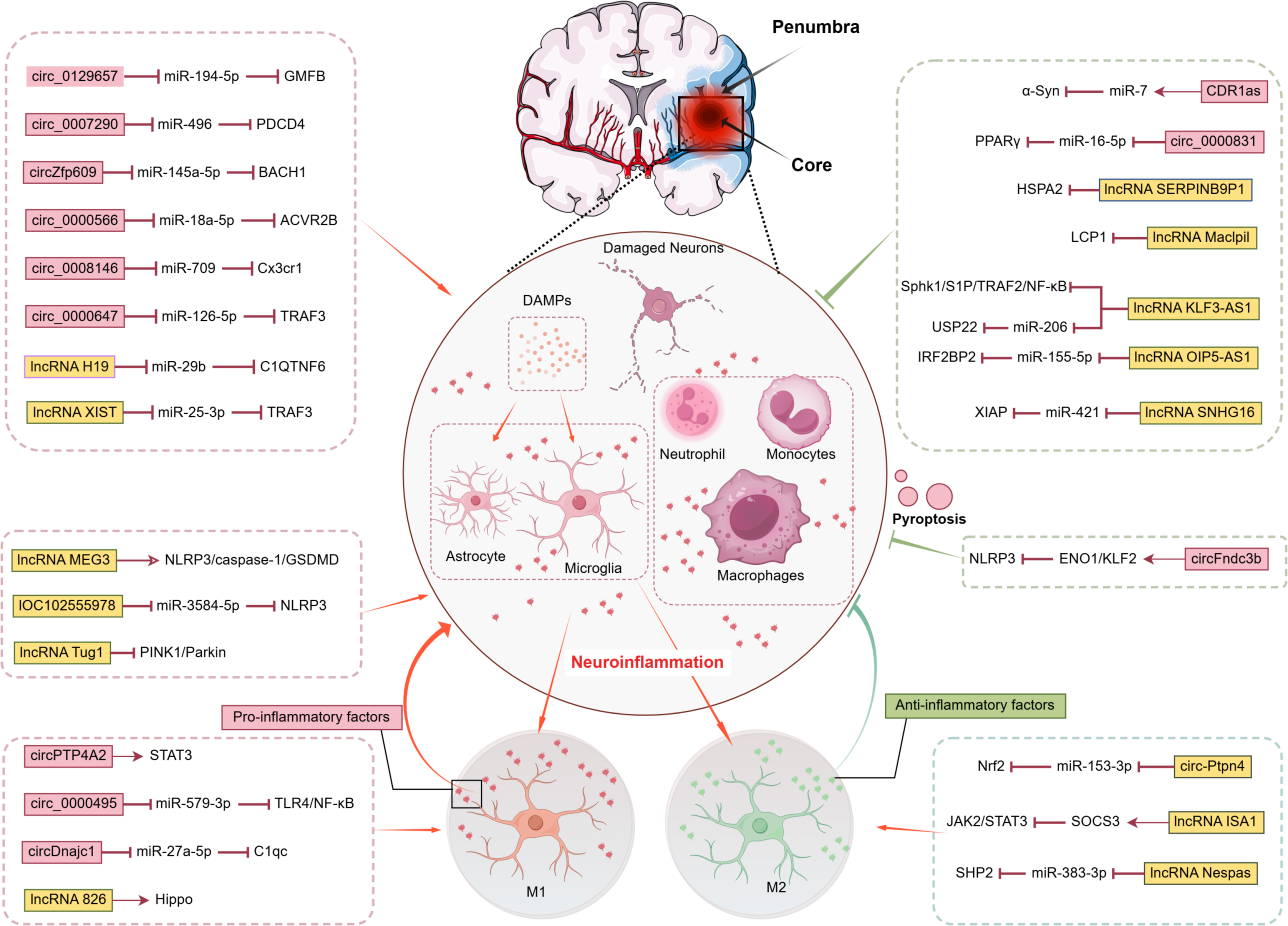
Post-Stroke Neuroinflammation Cascade Regulatory Network of CircRNAs and LncRNAs.

Reperfusion injury constitutes a multifactorial process involving sustained neuroinflammatory responses, oxidative stress, excitotoxicity, calcium overload, and their intricate interconnections, collectively exacerbating post-ischemic cerebral damage (16, 49, 50). The neuroinflammatory cascade in IS strictly fulfills the four diagnostic criteria of neuroinflammation while orchestrating a sophisticated interplay between tissue destruction and repair mechanisms. As summarized in **Figure 1**, this interplay is intricately regulated by a network of non-coding RNAs, particularly circRNAs and lncRNAs, which function through diverse mechanisms, including sponging microRNAs and directly interacting with signaling proteins, to precisely modulate key inflammatory pathways and cell death processes such as pyroptosis. Notably, the term “neuroinflammation” suffers from conceptual overgeneralization in practical applications. Its ambiguous definitional boundaries not only risk misinterpretation of pathological mechanisms but may also induce cognitive biases among novice researchers. To address this conceptual ambiguity, previous studies have systematically defined four diagnostic hallmarks based on peripheral inflammation parallels: microglial activation, brain-specific infiltration of peripheral immune cells, significant elevation of inflammatory mediators, and secondary neurodegeneration (51–53). These pathological processes may induce either necrosis or programmed apoptosis, with the specific mode determined by injury severity and neuronal metabolic status. In post-ischemic inflammatory responses, damaged and dying cells release damage-associated molecular patterns (DAMPs) that initiate immune activation and exert critical regulatory functions (54). These DAMPs precisely engage pattern recognition receptor clusters, including Toll-like receptor (TLR) families and scavenger receptors, on microglia and cerebrovascular endothelial cells. Functioning as biological radars within the sterile microenvironment devoid of exogenous pathogens, they breach the molecular silence of the BBB to activate inflammatory responses across neurovascular unit components: microglia initiate phagocytic programs, astrocytes release chemokines, and endothelial cells deploy adhesion molecules to establish molecular pathways for subsequent leukocyte transmigration (55–57).

### 2.3 Temporal progression of neuroinflammation and damage-repair homeostasis

In summary, inflammation is a recognized core regulatory axis in stroke pathology, functioning as an integral component of I/R-induced cascades. It persists throughout the entire pathological process from onset to post-injury repair, exhibiting dual regulatory characteristics of damage-repair homeostasis across different post-stroke phases (58, 59). This process can be divided into three stages:

1. Acute phase (initial hours post-stroke): Primarily characterized by phagocytic activity toward necrotic cells and debris, mediated jointly by resident phagocytes (microglia and macrophages) and infiltrating leukocytes (mainly neutrophils).
2. Subacute phase (days post-stroke): Marks gradual inflammation resolution.
3. Late phase (days to weeks post-stroke): Dominated by collaborative effects of astrocytes and microglia, leading to glial scar formation (60).

While glial scarring is essential for reconstructing vascular networks and sealing lesion areas to restore tissue integrity, it concurrently induces axonal regeneration barriers that significantly impair central nervous system functionality. Clinical studies demonstrate that elevated inflammatory levels correlate with poorer therapeutic outcomes in IS, establishing inflammation as a critical prognostic factor. For instance, early measurements of C-reactive protein (CRP) effectively predict functional disability and mortality in acute IS patients (61). The detrimental effects of inflammation extend beyond these observations. In focal and global cerebral I/R injury, intracranial hemorrhage, or mechanical brain trauma, inflammatory responses consistently exacerbate secondary damage despite incomplete mechanistic understanding (57). Current evidence suggests that targeted modulation of neuroinflammation may provide protective effects in IS, opening new therapeutic avenues. Rational immunomodulation could suppress pathological damage while enhancing reparative functions, though potential side effects require caution. Future research should clarify molecular mechanisms and signaling pathways underlying inflammatory regulation, precisely distinguish beneficial versus harmful targets, and develop safer therapeutic strategies to improve patient prognosis.

## 3 Limitations of conventional therapies for IS: breakthrough directions in ncRNA-targeted neuroinflammation

### 3.1 Temporal-spatial dilemmas and risk limitations of reperfusion therapies

The core contradiction in acute IS treatment lies in the spatiotemporal limitations of recanalization and associated therapeutic risks. Current strategies primarily include IVT and EVT.

#### 3.1.1 Rigid temporal window constraints

Alteplase (rt-PA), the only approved standard thrombolytic drug, must be administered within 4.5 hours of symptom onset. However, delayed hospital arrival severely limits its application. For example, only 12.43% of Chinese patients aged 65 or older arrive within 3 hours, with even lower rates in rural areas, compared to 40% to 60% in high-income countries (5, 62–66). Although next-generation thrombolytics, such as Tenecteplase and Reteplase, extend therapeutic windows through structural optimization and simplified administration, their clinical translation remains constrained by progressively increasing hemorrhagic risks, with intracranial hemorrhage risk higher than that of alteplase (64, 67, 68).

#### 3.1.2 Spatial accessibility imbalance

While EVT extends the recanalization window to 24 hours and improves outcomes in large-vessel occlusion patients, its implementation faces three barriers: (1) strict indications excluding small-vessel disease populations; (2) insufficient equipment and technical expertise at primary healthcare institutions; and (3) procedure-related vascular injury and post-reperfusion injury risks requiring multimodal imaging-guided patient selection (12, 69–72).

### 3.2 Precision balancing challenges in antithrombotic therapy

Antithrombotic therapy is a cornerstone of primary and secondary IS prevention, utilizing antiplatelet agents (e.g., aspirin, clopidogrel) or anticoagulants (e.g., warfarin, non-vitamin K antagonist oral anticoagulants [NOACs]) to suppress thrombogenesis (12). Treatment strategies are tailored to thrombotic etiology: antiplatelet regimens are favored for non-cardioembolic IS subtypes, such as atherosclerotic and small vessel disease (73, 74), while anticoagulation is preferred for cardioembolic origins, such as atrial fibrillation, or hypercoagulable states (75, 76). However, all antithrombotic approaches require a careful balance between ischemic protection and hemorrhagic risk, particularly gastrointestinal and intracranial hemorrhage. Effective clinical decision-making involves: Personalized bleeding risk assessment using validated scoring systems; Continuous monitoring of hemostatic parameters; and Prompt regimen adjustments based on the recurrence of thrombotic or hemorrhagic events. This delicate risk-benefit balance highlights the critical need for precision medicine in IS prophylaxis.

### 3.3 ncRNAs targeting neuroinflammation: molecular keys to overcoming spatiotemporal constraints

**Figure 2:**
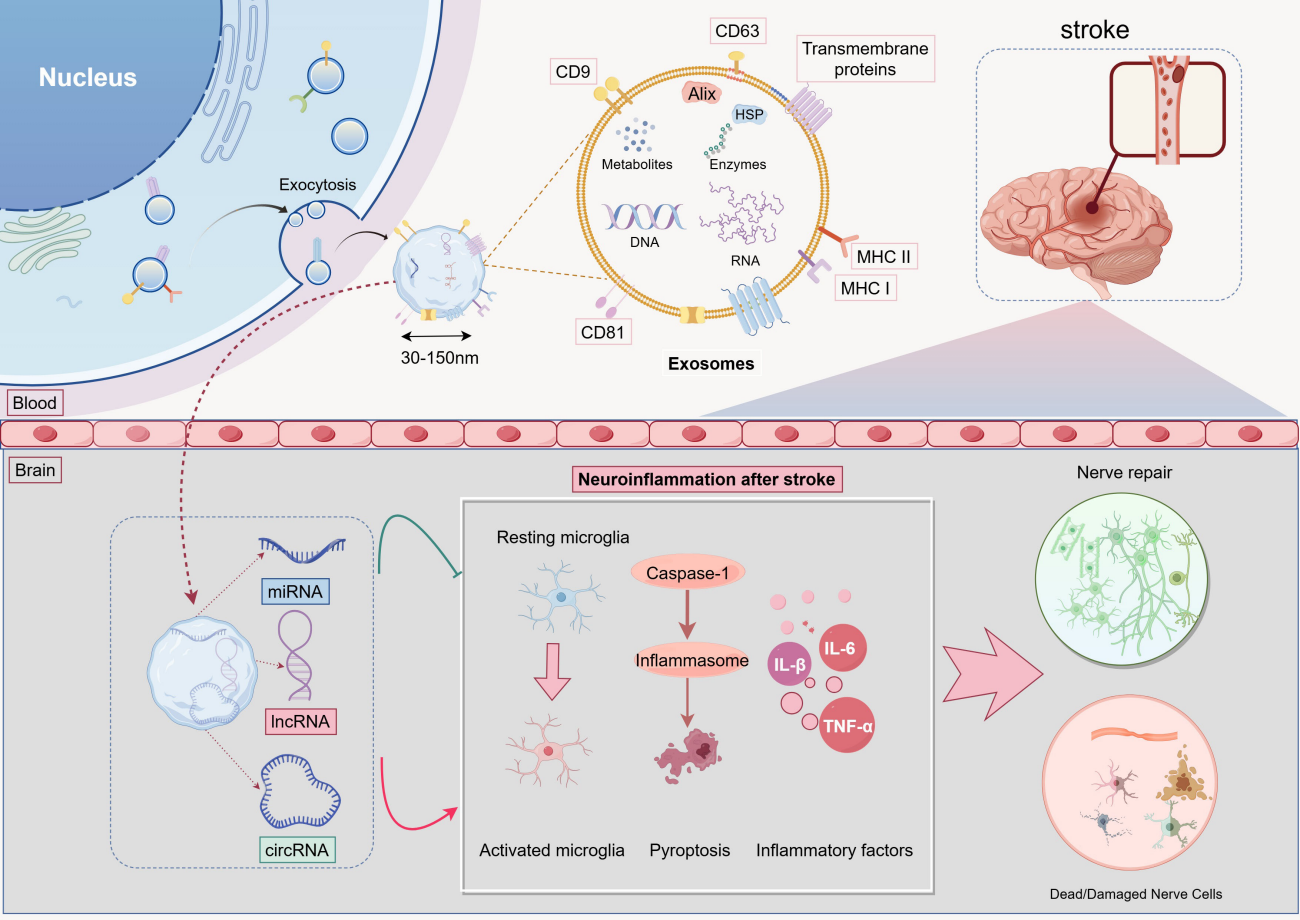
Exosomal ncRNAs Regulate Neuroinflammation After Stroke.

Current therapeutic strategies for IS emphasize rapid reperfusion through IVT and EVT, paired with etiology-guided antithrombotic management using antiplatelet or anticoagulant therapy. However, their clinical efficacy is limited by strict time windows, eligibility criteria, and an inability to mitigate neuroinflammation-driven secondary injury following reperfusion (77, 78). Increasing evidence positions inflammation as a central regulator in I/R cascades and a key mediator of risk factors. Notably, inflammatory processes compromise neurovascular unit (NVU) integrity prior to IS onset and continuously worsen secondary damage post-stroke (57, 79, 80). After reperfusion, aberrant neuroimmune responses initiate uncontrolled inflammatory cascades via mechanisms like inflammasome activation and mitochondrial dysfunction, resulting in BBB disruption, reactive oxygen species (ROS) bursts, and subsequent neuronal apoptosis and glial activation (81–83). Recent research identifies ncRNAs as critical regulators of post-reperfusion neuroinflammation, modulating neuroimmune interactions, suppressing inflammatory signaling pathways (e.g., NF-κB), and preserving NVU homeostasis (84). Their dynamic expression profiles, such as miR-124 and lncRNA MALAT1, not only act as early biomarkers of injury but also provide innovative therapeutic opportunities to overcome time window constraints through targeted delivery systems, such as exosomal vectors, facilitating precise anti-inflammatory interventions (32, 85–87). In this regard, stem cell-derived exosomes have emerged as highly effective ncRNA carriers, owing to their BBB permeability and biocompatibility. Experimental studies demonstrate their ability to deliver functional ncRNAs to ischemic lesions efficiently, maintaining bioactivity and coordinating molecular regulation in affected regions (88–90) (**Figure 2**). This advancement lays a theoretical groundwork for multidimensional strategies that integrate reperfusion, anti-inflammation, and neuroprotection, potentially overcoming the fundamental limitations of conventional IS therapies.

## 4 ncRNAs regulating neuroinflammation in IS

### 4.1 Regulatory roles of circRNAs in post-stroke neuroinflammatory cascades

Although members of the ncRNA family share common anti-neuroinflammatory functions, their mechanisms of action differ significantly (**Table 1**). As a key member of this family, circRNAs are covalently closed, single-stranded circular molecules formed through back-splicing of precursor RNAs (91). Based on their biogenesis mechanisms and structural features, circRNAs can be classified into four major subtypes: intron-derived ciRNAs (circular intronic RNAs), exon-intron chimeric EIciRNAs (exon-intron circRNAs), exon-cyclized ecircRNAs (exonic circRNAs), and tRNA-processing-derived tricRNAs (tRNA intronic circRNAs) (92, 93). Current studies reveal that circRNAs exhibit diverse functions, including acting as miRNA sponges, interacting with RNA-binding proteins (RBPs), regulating alternative splicing, transcription, and translation, generating pseudogenes, and participating in cellular transport and communication (93). In cerebral I/R injury, circRNAs primarily function as dynamic regulators of neuroinflammation. As evidenced by mechanistic studies, they not only directly modulate inflammatory responses but also critically sequester miRNAs through sponge activity to fine-tune inflammation levels, thereby steering neuroinflammatory progression (94, 95). The following representative examples illustrate this regulatory duality:

**Table 1:** Role of ncRNA in IS-associated neuroinflammatory responses.

ncRNA	Expression	Model/Diseases	Mechanism of Pyroptosis Regulation	Reference
CDR1as	↓	MCAO	Stabilizes miR-7 to suppress ​α-Syn toxicity, reducing apoptosis and neuroinflammation.	(96)
circ_0000831	↓	IS/MCAO/OGD	Sponges miR-16-5p, activates ​PPARγ signaling, and inhibits ​NF-κB-mediated inflammation.	(97)
circ_0129657	↑	OGD	Sponges miR-194-5p to upregulate ​GMFB, promoting inflammation and apoptosis.	(98)
circZfp609	↑	MCAO/OGD	Sponges miR-145a-5p to upregulate ​BACH1, exacerbating apoptosis and inflammation.	(99, 100)
circ_0007290	↑	AIS/OGD	Sponges miR-496 to upregulate ​PDCD4, promoting neuronal apoptosis and inflammation.	(101)
circ_0000566	↑	OGD/R	Sponges miR-18a-5p to upregulate ​ACVR2B, driving inflammation, apoptosis, and vascular injury.	(102)
circ_0008146	↑	MCAO/LPS	Sponges miR-709 to activate ​Cx3cr1 signaling, promoting IL-6 and TNF-α release and exacerbating neuroinflammation.	(103)
circ_0000647	↑	OGD/R	Sponges miR-126-5p to upregulate ​TRAF3, accelerating apoptosis, inflammation, and oxidative stress.	(104)
lncRNA Maclpil	↓	MCAO	Knockdown inhibits ​LCP1, impairing macrophage migration and pro-inflammatory polarization.	(111)
lncRNA KLF3-AS1	↓	MCAO/ OGD/R	Sponges miR-206 to stabilize ​USP22/Sirt1 (inhibiting NF-κB) or suppresses ​Sphk1/S1P/TRAF2/NF-κB signaling.	(112, 113)
lncRNA OIP5-AS1	↓	OGD/R	Sponges miR-155-5p to derepress ​IRF2BP2, attenuating oxidative stress and inflammation.	(114)
lncRNA SERPINB9P1	↓	IS/OGD/R	Binds ​HSPA2 to suppress IL-6 and TNF-α, upregulates ​Bcl-2/Bax ratio, and alleviates ischemia-reperfusion injury.	(115)
lncRNA SNHG16	↓	OGD/R	Sponges miR-421 to upregulate ​XIAP, inhibiting caspase activity and reducing apoptosis/inflammation.	(116)
lncRNA H19	↑	MCAO/OGD	Directly binds miR-29b to upregulate ​C1QTNF6, enhancing pro-inflammatory factor release.	(117)
lncRNA XIST	↑	OGD/R	Sponges miR-25-3p to upregulate ​TRAF3, promoting pro-inflammatory cytokine release and apoptosis.	(118)
miR-193a-5p	↓	AIS、MCAO、LPS	Inhibits ​UBE2V2 to stabilize ​PPARγ, promoting neutrophil polarization toward anti-inflammatory phenotypes	(123)
miR-369-3p	–	MCAO/R、OGD/R	Downregulates ​PDE4D to activate ​cAMP/PKA/AMPK signaling, suppressing inflammation and oxidative stress.	(124)
miR-6328	↑	MCAO/R、OGD/R	Suppresses ​IKKβ/NF-κB signaling, reducing IL-1β and TNF-α release.	(125)
miR-124-5p	↓	MCAO/R、OGD	Targets ​FoxO1 to inhibit inflammation and apoptosis.	(126)
miR-30c-5p	↓	OGD/R、MCAO/R	Inhibits ​GNAI2/NF-κB signaling, reducing IL-1β, IL-6, and TNF-α.	(127)
miR-182-5p	–	MCAO/R、OGD/R	Downregulates ​Rac1/NF-κB/NOX2 signaling to inhibit astrocyte inflammation.	(128)
miR-149	↓	MCAO	Directly binds ​TNF-α/IL-6 mRNAs to block translation, protecting the blood-brain barrier.	(129)
miR-15a/16-1	↑	MCAO	Promotes apoptosis, inflammation, oxidative stress, and suppresses angiogenesis.	(130)
miR-30c-2-3p	↑	AIS、tMCAO	Inhibits ​SMAD2 to block TGF-β anti-inflammatory effects, promoting IL-6 and TNF-α release.	(131, 132)
miR-Novel-3	↑	LAA-AIS、MCAO、OGD	Targets ​STRN to inhibit ​PI3K-AKT-mTOR signaling, driving microglial ferroptosis.	(133)
miR-9-5p	↑	LPS	Downregulates miR-9-5p to reduce IL-1β, IL-6, iNOS, and TNF-α in microglia.	(134)
miR-188-5p	↑	MCAO/R、OGD/R	Targets ​Lin28a to exacerbate apoptosis and inflammation.	(135)

↑ : Upregulation | ↓: Downregulation | —: Not mentioned.

CDR1as/ciRS-7 exemplifies a unique circRNA-mediated neuroprotective mechanism. Enriched with miR-7 binding sites, it stabilizes miR-7 via incomplete complementary pairing, extending its biological half-life. This interaction suppresses α-synuclein (α-Syn), a key regulator of the TLR4/NF-κB pathway, ultimately attenuating neuroinflammation, reducing neuronal apoptosis, and enhancing motor function recovery in ischemic models (96). circ_0000831 represents another pivotal anti-inflammatory circRNA, characterized by its downregulation in stroke patients. By serving as a molecular sponge for miR-16-5p, it activates adiponectin receptor 2 (AdipoR2) and the PPARγ signaling pathway, significantly alleviating neuroinflammation, vertigo, neurological deficits, and apoptosis in MCAO mice. Dual-luciferase assays confirm its direct binding to miR-16-5p, solidifying its mechanistic role (97).

Conversely, a distinct class of pro-inflammatory circRNAs exacerbates ischemic injury. For example, circ_0129657 competitively binds miR-194-5p, relieving its suppression of glia maturation factor beta (GMFB). This derepression promotes endothelial apoptosis and inflammatory cytokine secretion, while its silencing reduces cerebral infarct volume and ameliorates neurological dysfunction (98). circZfp609 mediates damage through a dual regulatory network: it serves as a sponge for miR-145a-5p both to suppress NF-κB inhibition and upregulate BACH1, thereby promoting apoptosis and pro-inflammatory factor release (e.g., IL-1β, TNF-α). Genetic knockout of circZfp609 alleviates cerebral ischemia/reperfusion injury and inhibits OGD/R-induced cellular damage by modulating astrocyte apoptosis and inflammatory responses (99, 100). Other pro-inflammatory circRNAs—including circ_0007290 (via miR-496/PDCD4), circ_0000566 (targeting miR-18a-5p), circ_0008146 (miR-709/CX3CR1 axis), and circ_0000647 (miR-126-5p/TRAF3)—likewise function as miRNA sponges to amplify ROS bursts, cytokine secretion, and endothelial injury (101–104).

In summary, circRNAs critically orchestrate neuroinflammatory responses post-stroke, primarily through sponge-mediated miRNA regulation. While anti-inflammatory members (e.g., CDR1as, circ_0000831) stabilize protective miRNAs to suppress inflammatory cascades, their pro-inflammatory counterparts (e.g., circ_0129657, circZfp609) sequester repressive miRNAs to activate apoptotic and inflammatory pathways. This functional duality positions circRNAs as promising therapeutic targets for modulating post-stroke neuroinflammation.

### 4.2 LncRNAs orchestrate neuroimmune crosstalk after stroke

LncRNAs are non-coding RNA molecules exceeding 200 nucleotides (nt) in length. Thousands of lncRNAs have been identified in the human genome, regulating gene expression through chromatin modification and post-transcriptional regulatory mechanisms (105–107). LncRNA dysregulation is closely associated with pathological processes in various diseases. The central nervous system harbors the most extensive repertoire of lncRNA transcripts, which participate in multidimensional regulation of neural development and homeostasis maintenance. Studies using microarray and high-throughput RNA sequencing technologies have identified hundreds of differentially expressed lncRNAs in IS patients and animal models. These expression profile abnormalities are strongly correlated with pathological processes such as BBB disruption and neuroinflammation (108–110).

At the neuroimmune interface, lncRNA Maclpil exhibits unique properties by blocking monocyte-derived macrophage (MoDM) activation and migration through inhibition of LCP1-mediated actin dynamics. Crucially, LCP1 knockout fully replicates Maclpil’s neuroprotective effects, confirming LCP1 as its key effector. Consistent with this, animal studies demonstrate that this axis reduces infarct volume and improves neurological recovery (111). Similarly, KLF3-AS1 stands out for its dual regulatory mechanisms. In cerebral I/R models, its downregulation correlates with elevated pro-inflammatory factors (TNF-α, IL-6, IL-1β). Mechanistically, KLF3-AS1 suppresses neuroinflammation via the Sphk1/NF-κB pathway (112). Furthermore, when delivered by BMSC-derived exosomes, it competitively binds miR-206 to upregulate USP22, promoting Sirt1 deubiquitination and extending its half-life to mitigate inflammation and apoptosis (113). Other protective lncRNAs (e.g., OIP5-AS1, SERPINB9P1, SNHG16) similarly modulate ceRNA networks, chaperone activity, and oxidative pathways to mitigate injury (114–116).

Conversely, some lncRNAs drive pathology. lncRNA H19 demonstrates significant clinical relevance, with its expression in neutrophils of IS patients positively correlating with leukocyte activation. Mechanistically, H19 acts as a ceRNA for miR-29b, relieving its inhibition of C1QTNF6 and driving pro-inflammatory factor release (IL-1β, TNF-α), thereby exacerbating BBB disruption and ischemic damage (117). Similarly, lncRNA XIST exhibits striking cell-type specificity in neurons. It sponges miR-25-3p to elevate TRAF3 levels, activating NF-κB-mediated inflammatory responses. Supporting its detrimental role, XIST silencing inhibits neuronal apoptosis and cytokine release, while exogenous TRAF3 overexpression reverses this protection (118).

In summary, as lncRNAs bridge epigenetic regulation and immune dynamics, they represent not just therapeutic targets but potential “molecular switches” for reprogramming neuroinflammation, offering a promising platform for developing lncRNA-mediated precision medicine strategies in stroke recovery.

### 4.3 miRNAs: precision modulators of post-stroke inflammatory microenvironment

MiRNAs are small non-coding RNA molecules approximately 20–24 nucleotides (nt) in length. They exert critical post-transcriptional regulatory roles by mediating target mRNA degradation or translational repression through incomplete base complementarity. Notably, a single miRNA can regulate the translation of hundreds of mRNAs, demonstrating its broad functional regulatory potential (119–122). Given their ability to directly target key inflammatory factor mRNAs, miRNAs have become a research focus in I/R injury in recent years. For example, multiple studies confirm that targeting specific miRNAs effectively modulates I/R-associated neuroinflammatory cascades, offering novel therapeutic avenues for IS.

Specific examples highlight this therapeutic potential. miR-193a-5p, for example, orchestrates neutrophil immune polarization. In acute IS patients, its significant downregulation in circulating neutrophils disrupts PPARγ-mediated anti-inflammatory signaling. Mechanistically, miR-193a-5p targets UBE2V2 to inhibit PPARγ ubiquitination, promoting N2 phenotype polarization and reducing pro-inflammatory cytokine release. Importantly, rescue experiments confirm that UBE2V2 knockdown reverses neutrophil hyperactivation in miR-193a-5p-deficient models (123). Recent advances in exosome delivery strategies have opened new avenues for miRNA-based therapies. For instance, exosomes derived from recombinant human growth differentiation factor 7 (rhGDF7)-preconditioned bone marrow mesenchymal stem cells (BMSCs) are enriched with miR-369-3p. This miRNA targets the 3’-untranslated region (3’-UTR) of the *Pde4d* gene, suppressing PDE4D protein expression and activating the AMPK signaling pathway, thereby significantly inhibiting I/R-induced neuronal inflammation and oxidative stress damage (124). Similarly, miR-6328 exerts critical anti-inflammatory effects in I/R injury by targeting the IKKβ/NF-κB signaling axis. Mechanistically, this miRNA binds to IKKβ to inhibit its expression, thereby blocking NF-κB pathway activation. Notably, sequencing at 72 hours post-I/R revealed elevated miR-6328 levels accompanied by reduced IKKβ, and reverse functional experiments further validated the specificity of its anti-inflammatory effects (125). In addition to these, other protective miRNAs (e.g., miR-124-5p, miR-30c-5p, miR-182-5p, miR-149) orchestrate anti-inflammatory responses primarily by suppressing neuronal apoptosis and reinforcing BBB integrity (126–129).

However, miRNA function is not context-independent. A prominent example underscoring the critical, yet often overlooked, role of age and sex factors in RNA biology is miR-15a/16-1. Significantly upregulated post-stroke and correlating with neuronal injury severity, studies in tMCAO models reveal a striking age-sex interaction: young female mice (3-month-old) exhibit the strongest neuroprotective phenotype, characterized by reduced miR-15a/16–1 induction. Combined antagonism with estradiol synergistically downregulates genes driving neurovascular death, inflammation, and oxidative stress. miR-15a/16–1 target genes further display marked age- and sex-dependent expression, solidifying their biomarker potential for tailored stroke therapy (130). Crucially, while most preclinical stroke studies utilize healthy rodent models, translating findings to human patients requires attention to human-specific factors. Recent investigations focusing on patient-derived biomarkers reveal critical mechanisms specific to human pathology. For instance, circulating miR-30c-2-3p is uniquely upregulated in plasma exosomes of acute ischemic stroke (AIS) patients. It establishes a pro-inflammatory network by targeting SMAD2, a core anti-inflammatory component of the TGF-β pathway, suggesting exosome-mediated miR-30c-2-3p delivery as a key driver of post-stroke neuroinflammation (131, 132). Notably, research delving into specific stroke subtypes uncovers further complexity. Research on the large artery atherosclerotic (LAA) stroke subtype identifies plasma exosome-derived miR-Novel-3 as a dual pathogenic factor. Specifically overexpressed in LAA-AIS patients, it concurrently drives neuroinflammation and ferroptosis by targeting STRN protein in peri-infarct microglia/macrophages, thereby suppressing PI3K-AKT-mTOR signaling to trigger inflammatory mediator release and ferroptosis-related molecular dysfunction, ultimately exacerbating ischemic neuronal injury (133).These findings collectively highlight the necessity of incorporating patient-specific complications (e.g., atherosclerosis) into stroke models to uncover clinically relevant pathological cascades. Additional detrimental miRNAs (e.g., miR-9-5p, miR-188-5p) exacerbate neuroinflammation through dysregulated TLR signaling, driving maladaptive pro-inflammatory cascades (134, 135).

## 5 ncRNA regulation of microglial polarization in IS

### 5.1 Molecular basis of microglial polarization

Microglia originate from erythromyeloid progenitors (EMPs) in the early embryonic yolk sac. Their development occurs independently of the hematopoietic stem cell-mediated system, culminating in their differentiation into tissue macrophages resident in the brain parenchyma (136–138). As the central nervous system’s primary immune effector cells, microglia constitute approximately 10% of total neural cells and dynamically maintain neuroimmune homeostasis to support brain function (139–141).

In IS, microglia display two distinct polarization states:

1. Pro-inflammatory phenotype (M1 polarization): Microglia detect DAMPs through pattern recognition receptors (PRRs), initiating neuroinflammatory cascades and releasing pro-inflammatory cytokines, such as TNF-α and IL-1β. This response disrupts the BBB and amplifies neuronal death (142–145).
2. Anti-inflammatory phenotype (M2 polarization): Microglia foster neurogenesis, angiogenesis, and synaptic remodeling by clearing apoptotic debris via phagocytosis and secreting brain-derived neurotrophic factor (BDNF), thus preserving microenvironmental homeostasis (146, 147).

In stroke, excessive DAMP stimulation skews the M1/M2 balance toward the pro-inflammatory M1 phenotype. Encouraging M2 polarization can mitigate inflammation and enhance tissue repair, improving post-stroke outcomes (53, 148). Consequently, modulating microglial polarization—particularly toward the M2 state—represents a key strategy for reducing I/R injury. Among these approaches, ncRNA-mediated regulation stands out for its precision and capacity to target multiple pathways simultaneously (**Table 2**).

**Table 2:** ncRNA-mediated microglial regulation in Post-IS inflammation.

ncRNA	Expression	Model/Diseases	Mechanism of Pyroptosis Regulation	Reference
circ-Ptpn4	–	MCAO、OGD	Acts via ​miR-153-3p/Nrf2 axis to drive M2 polarization.	(149)
lncRNA ISA1	↓	OGD/R、MCAO	Upregulates ​SOCS3 to inhibit ​JAK2/STAT3 pathway, promoting M2 polarization.	(150)
lncRNA Nespas	–	MCAO、OGD/R	Sponges ​miR-383-3p to derepress ​SHP2, inhibiting ​NLRP3 inflammasome and driving M2 polarization.	(151)
miR-124-3p	↓	OGD、MCAO	Suppresses ​TRAF6/NF-κB axis, driving M2 polarization.	(152)
miR-103-3p	↓	OGD/R、MCAO	Enhances ​TANK expression to inhibit ​NF-κB-mediated microglial activation.	(153)
circPTP4A2	↑	tMCAO、OGD/R	Binds ​STAT3 to promote its phosphorylation, driving microglial M1 polarization and pro-inflammatory cytokine release (TNF-α, IL-1β↑)	(154)
circ_0000495	↑	OGD、MCAO	Exosomal transfer to microglia activates ​miR-579-3p/TLR4/NF-κB axis, inducing M1 polarization.	(155)
circDnajc1	↑	OGD/R、MCAO/R	Sponges ​miR-27a-5p to upregulate ​C1qc, activating ​C1qc/C3/C5aR complement pathway and microglial activation.	(156)
lncRNA 826	↑	OGD、MCAO	Activates ​Hippo pathway to induce YAP/TAZ phosphorylation and degradation, driving M1 polarization.	(157)
lncRNA NEAT1	–	LPS	Inhibits ​AMPK pathway and activates ​NF-κB, promoting M1 polarization and suppressing angiogenesis.	(158)
miR-210	↑	MCAO	Targets ​TET2 (via 3'UTR binding) to promote microglial activation and pro-inflammatory cytokine release.	(159)
miR-100-5p	↑	MCAO、OGD/R	Delivered via EVs to microglia, activates ​TLR7/NF-κB pathway and pro-inflammatory responses.	(160)

↑ : Upregulation | ↓: Downregulation | —: Not mentioned.

### 5.2 ncRNA-mediated regulation of microglial polarization

#### 5.2.1 ncRNAs promote anti-inflammatory polarization and neural repair

Recent advancements in exosome delivery systems have introduced innovative strategies to modulate microglial polarization. For example, exosomes from hypoxia-preconditioned adipose-derived mesenchymal stem cells (ADSCs) deliver circ-Ptpn4, which significantly promotes microglial polarization toward the anti-inflammatory M2 phenotype. Mechanistic studies demonstrate that circ-Ptpn4 acts as a sponge for a specific miRNA, thereby relieving its transcriptional repression of nuclear factor erythroid 2-related factor 2 (Nrf2). This interaction activates antioxidant and anti-inflammatory signaling pathways, ultimately reducing neuronal death (149). These findings confirm the role of exosomes as effective carriers and underscore the pivotal contribution of circRNAs to phenotypic reprogramming.

The lncRNA ISA1 is a critical regulator. In MCAO mouse models, decreased ISA1 expression correlates with larger cerebral infarct volumes and greater neurological deficits. Overexpression of ISA1 exerts neuroprotective effects by activating suppressor of cytokine signaling 3 (SOCS3), which inhibits the JAK2/STAT3 signaling pathway. This suppression drives microglial polarization toward the anti-inflammatory phenotype (150). Equally important in MCAO is the role of ceRNA networks. In MCAO models, elevated miR-383-3p levels exacerbate injury by activating NLR family pyrin domain-containing 3 (NLRP3) inflammasome activity. The lncRNA Nespas mitigates this effect by sponging miR-383-3p, which relieves its inhibition of Src homology 2 domain-containing protein tyrosine phosphatase 2 (SHP2). This establishes the Nespas/miR-383-3p/SHP2 regulatory axis, offering protective effects through three mechanisms:

1. Suppressing NLRP3 inflammasome assembly;
2. Upregulating the M2 marker interleukin-10 (IL-10); and
3. Downregulating M1-associated factors, such as tumor necrosis factor-alpha (TNF-α) and inducible nitric oxide synthase (iNOS).

Together, these actions mitigate neuroinflammatory progression (151).

MiRNA regulatory networks also provide precise modulation of polarization. In OGD models, decreased miR-124-3p levels result in overactivation of the TRAF6/NF-κB signaling pathway. Supplementing with exogenous miR-124-3p inhibits TRAF6-mediated NF-κB phosphorylation, promoting microglial polarization toward the M2 phenotype and enhancing neuronal survival (152). Similarly, in MCAO rat models, miR-103-3p targets the 3’-untranslated region (3’-UTR) of TANK mRNA, thereby modulating the NF-κB signaling pathway. This regulation suppresses abnormal microglial activation, alleviating I/R injury (153).

#### 5.2.2 ncRNAs drive pro-inflammatory polarization and neuroinflammatory injury

In contrast to their anti-inflammatory counterparts, certain ncRNAs exacerbate neuroinflammatory damage post-ischemic stroke by driving microglial polarization toward the pro-inflammatory M1 phenotype through regulation of key signaling pathways.

Within pro-inflammatory circRNA networks, circPTP4A2 serves as a representative example. In tMCAO models, its expression is markedly upregulated in plasma and peri-ischemic cortical tissues. Functional studies demonstrate that circPTP4A2 knockout significantly ameliorates cerebral injury in multiple ways: reducing infarct volume, restoring cortical blood flow homeostasis, and alleviating neurological deficits. Mechanistically, circPTP4A2 directly binds to STAT3 protein to enhance its phosphorylation levels, driving microglial M1 polarization while suppressing M2 phenotypic transition. *In vitro* validation confirms that circPTP4A2 silencing in OGD/R-induced BV2 microglia significantly inhibits STAT3 signaling activation, decreases pro-inflammatory factor release (TNF-α, IL-6), and increases anti-inflammatory mediator expression (154). Another key circRNA, circ_0000495, is transferred from OGD-treated human brain microvascular endothelial cells (HBMVECs) to microglia via exosomes. It competitively binds miR-579-3p to relieve its suppression of the TLR4/NF-κB pathway. This cascade significantly elevates M1 markers (TNF-α, IL-1β, IL-12) and CD86+ cell proportions while suppressing M2 markers (IL-10, CD163), ultimately exacerbating endothelial damage (155). In another pathological mechanism involving complement system activation, hypoxia-induced circDnajc1 accumulation during I/R injury sponges miR-27a-5p to alleviate its inhibition of complement component C1qc. This triggers uncontrolled activation of the complement cascade (C3/C5aR axis), driving microglial M1 polarization, promoting inflammatory mediator release, and disrupting the BBB. Targeted inhibition of circDnajc1 effectively blocks neuroinflammatory propagation (156).

Certain lncRNAs also drive pro-inflammatory activation. For example, Lnc826 is significantly upregulated in MCAO models. It selectively enhances M1 marker expression (TNF-α, IL-1β) while suppressing M2 markers (e.g., IGF-1) by modulating the Hippo signaling pathway. Notably, Lnc826 knockdown in OGD-induced cellular models reverses microglial pro-inflammatory polarization by restoring the activity of YAP, a downstream effector of the Hippo pathway (157). The pro-inflammatory effects of lncRNA NEAT1 are linked to energy metabolism dysregulation. In BV-2 microglia, NEAT1 inhibits AMPK phosphorylation, reduces SIRT1 activity, and relieves NF-κB pathway suppression, thereby driving M1 polarization. Functional experiments demonstrate that NEAT1 overexpression significantly impairs tube formation capacity in mouse cerebral arterial endothelial cells (mCAECs), while AMPK activators or NEAT1 silencing restore angiogenic activity and promote M2 polarization (158).

MiRNAs also play a critical role in exacerbating neuroinflammation. MicroRNA-210 (miR-210) exacerbates neuroinflammation through epigenetic modifications. It suppresses TET2 expression by targeting its 3’-UTR, amplifying neuroinflammatory responses post-ischemic stroke. Experimental studies demonstrate that miR-210 mimics significantly reduce TET2 levels in murine brains, whereas miR-210 knockout or antagonist interventions maintain TET2 expression. TET2 inhibits pro-inflammatory cytokines via NF-κB pathway regulation: TET2 overexpression reverses miR-210-induced elevations in IL-6/IL-1β. In stroke models, miR-210 upregulation suppresses TET2, directly driving microglial activation and pro-inflammatory polarization, as evidenced by increased CD11b+ microglia and IL-6 release. TET2 knockdown completely negates the neuroprotective effects of miR-210 inhibition, including reduced infarct volume and improved neurological function (159). Furthermore, exosome-mediated neuron-microglia communication plays a pivotal role in post-stroke neuroinflammation. Ischemia-induced neuronal hyperactivity releases sEVs enriched with miR-100-5p, which are internalized by neighboring neurons and microglia via paracrine signaling. Mechanistically, miR-100-5p directly binds Toll-like receptor 7 (TLR7) through its unique U18U19G20 motif, activating NF-κB phosphorylation cascades. This triggers explosive release of pro-inflammatory factors (IL-1β, TNF-α) and suppresses the anti-inflammatory marker arginase-1 (Arg-1). In MCAO model mice, miR-100-5p levels are markedly elevated in peri-ischemic microglia-derived sEVs. Intracerebroventricular injection of miR-100-5p antagomiR specifically silences this miRNA, effectively blocking TLR7/NF-κB signaling, reversing microglial pro-inflammatory activation, and significantly reducing infarct volume while improving motor function outcomes (160).

## 6 ncRNA regulation of pyroptosis in IS: linking inflammation and neuronal death

### 6.1 Pathological mechanisms of pyroptosis and its association with stroke

Pyroptosis, an inflammatory form of programmed cell death mediated by Gasdermin proteins, is characterized by plasma membrane rupture and the release of pro-inflammatory factors (161, 162). Unlike apoptosis, which maintains membrane integrity, pyroptosis induces the efflux of cytoplasmic inflammatory contents through membrane pores, culminating in secondary necrosis—a pathological state of altered membrane permeability following complete cell death (161, 163–165).

The Gasdermin protein family comprises six members (GSDMA, GSDMB, GSDMC, GSDMD, GSDME, and GSDMF/PJVK/DFNB59), serving as core executors of inflammatory responses and disease progression (166).

Pyroptosis is closely linked to the initiation and progression of stroke. Post-stroke, Gasdermin-mediated pyroptosis displays cascading amplification effects within the neurovascular unit (167, 168). When pyroptosis is activated in neuroglia, neurons, and brain microvascular endothelial cells (BMECs), membrane perforation leads to the explosive release of inflammatory mediators like IL-1β and IL-18. This release generates self-amplifying cytokine storms (169, 170). This pathology exacerbates brain injury through two primary mechanisms. First, pyroptosis disrupts the physical barrier by dismantling the tight junction structures of the BBB, leading to plasma protein leakage and the infiltration of toxic substances into the brain parenchyma. Second, it causes molecular signaling dysregulation, where extracellular DAMPs activate microglia via pattern recognition receptors such as TLRs. This activation drives the spread of neuroinflammation to the ischemic penumbra, accelerating the conversion of reversible injury zones into infarct cores (171–174).

Recent advances in understanding the role of pyroptosis in stroke pathophysiology have shown that ncRNAs regulate pyroptosis signaling pathways at multiple levels by targeting critical components such as NLRP3 inflammasome activation and Caspase-1 processing (**Table 3**). This complex epigenetic regulation provides new insights into disrupting the vicious cycle of pyroptosis, neuroinflammation, and tissue.

**Table 3:** IS-associated pyroptotic signaling via ncRNA.

ncRNA	Expression	Model/Diseases	Mechanism of Pyroptosis Regulation	Reference
circFndc3b	↓	OGD/R、MCAO	Activates ​ENO1/KLF2 axis to inhibit ​NLRP3 inflammasome-mediated pyroptosis.	(175)
lnc OIP5-AS1	↓	IS、OGD/R、MCAO/R	Degrades ​TXNIP to block ​NLRP3 inflammasome activation, inhibiting neuronal pyroptosis.	(176)
miR-378a-5p	–	OGD、MCAO	Targets ​NLRP3-Caspase-1-GSDMD axis to suppress pyroptosis.	(177)
miR-96-5p	↓	OGD、MCAO	Inhibits ​caspase-1 to block pyroptosis cascade (caspase-1 → Gsdmd → membrane pore formation).	(178)
lncRNA MEG3	↑	OGD/R、MCAO/R	Activates ​NLRP3/caspase-1/GSDMD signaling to induce neuronal pyroptosis and cytokine release.	(179)
LOC102555978	↑	OGD/R、MCAO/R	Sponges ​miR-3584-5p to upregulate ​NLRP3, activating pyroptosis pathway.	(180)
lncRNA Tug1	↑	OGD/R	Suppresses ​PINK1/Parkin-mediated mitophagy, leading to mitochondrial damage and NLRP3 activation.	(181)

↑ : Upregulation | ↓: Downregulation | —: Not mentioned.

### 6.2 ncRNA-mediated pyroptosis control

#### 6.2.1 ncRNAs inhibiting pyroptosis

Recent studies highlight the neuroprotective potential of ncRNAs through targeted regulation of pyroptosis-related molecules.

For example, the expression of circFndc3b is inversely correlated with neuropathological severity in IS models. In MCAO models, circFndc3b levels are significantly downregulated in peri-infarct cortical regions, while exercise intervention upregulates circFndc3b to reduce infarct volume and enhance neurological recovery. Mechanistically, circFndc3b directly binds to enolase 1 (ENO1) and coordinates a dual-pathway regulation: First, it enhances ENO1-mediated stabilization of Klf2 mRNA via 3’-UTR interactions, thereby suppressing NLRP3 inflammasome-driven pyroptosis; second, it promotes ENO1-dependent stabilization of FUS mRNA to reinforce circFndc3b self-circularization, forming a self-reinforcing positive feedback loop. This mechanism not only identifies exercise intervention as a potential therapeutic target but also elucidates how circFndc3b maintains cellular homeostasis, offering critical theoretical support for post-stroke neurorestorative strategies (175).

Additionally, lncRNAs regulate pyroptosis. For example, exosome-delivered lncRNA OIP5-AS1 suppresses pyroptosis. In OGD/R-treated neurons and MCAO/R models, OIP5-AS1 levels are significantly downregulated, whereas exogenous supplementation with OIP5-AS1 reduces infarct volume and inhibits the expression of pyroptosis-related proteins. Mechanistic studies reveal that OIP5-AS1 directly binds to thioredoxin-interacting protein (TXNIP), promoting E3 ubiquitin ligase ITCH-mediated ubiquitination and degradation of TXNIP. This process blocks NLRP3 inflammasome activation and subsequent pyroptosis cascades (176).

MiRNAs also play pivotal roles in suppressing pyroptosis. Astrocyte-derived exosomes deliver miR-378a-5p to exert anti-pyroptotic effects. In OGD and MCAO models, miR-378a-5p binds to the 3’-UTR of the NLRP3 gene, inhibiting its transcription and inflammasome assembly, thereby significantly reducing pyroptosis marker levels. Functional studies confirm that miR-378a-5p expression is positively correlated with neuronal survival rates. Conversely, genetic knockout of miR-378a-5p induces dose-dependent upregulation of NLRP3 expression, exacerbating neuronal injury (177). Another anti-pyroptotic miRNA, miR-96-5p, suppresses pyroptosis by targeting the CASP1 gene rather than the broader caspase-1/GSDMD axis. In I/R models, decreased miR-96-5p expression is negatively correlated with activation of pyroptosis-related proteins (cleaved caspase-1, Gsdmd-N). Functional studies demonstrate that miR-96-5p directly binds to the CASP1 gene to inhibit its transcription. In OGD/R models, miR-96-5p mimics reduce TUNEL-positive cell ratios and improve neuronal survival. Animal experiments further validate these findings: intracerebroventricular delivery of miR-96-5p agomir downregulates the expression of pyroptosis executioner proteins in the ischemic penumbra, while also alleviating behavioral deficits and cerebral edema (178).

#### 6.2.2 ncRNAs promoting pyroptosis

ncRNAs exacerbate post-ischemic pyroptosis through diverse regulatory mechanisms. For example, lncRNA MEG3 promotes neuronal pyroptosis in cerebral I/R injury through epigenetic regulation. Ischemia-induced downregulation of the fat mass and obesity-associated protein (FTO) increases m6A methylation of MEG3, enhancing its stability and leading to pathological accumulation. This elevated MEG3 activates the NLRP3/caspase-1/GSDMD signaling pathway, triggering pyroptosis, as demonstrated by the concurrent upregulation of NLRP3, cleaved caspase-1, and GSDMD-N in cortical tissues. Importantly, overexpressing FTO reverses the aberrant m6A modification of MEG3 and suppresses pyroptosis, highlighting the FTO-MEG3 axis as a potential therapeutic target for stroke intervention (179).

Additionally, the ceRNA mechanism contributes to pyroptosis activation. In I/R models, lncRNA LOC102555978 is significantly upregulated and acts as a molecular sponge by competitively binding miR-3584-5p. This binding relieves the miRNA’s inhibition of NLRP3, thereby activating pyroptosis cascades. Disrupting the LOC102555978/miR-3584-5p/NLRP3 axis reduces pyroptosis markers, improves cell viability, and mitigates pathological damage, as shown in experimental studies (180).

Moreover, disrupting mitochondrial homeostasis exacerbates pyroptosis. Under hypoxic conditions, lncRNA Tug1 suppresses PINK1/Parkin-mediated mitophagy, thereby promoting microglial pyroptosis. Knocking down Tug1 activates the PINK1-Parkin pathway, as evidenced by an increased LC3B-II/LC3B-I ratio and enhanced p62 degradation. This activation enhances Parkin recruitment to mitochondria, facilitating the clearance of damaged mitochondria and inhibiting NLRP3 inflammasome activation. Notably, this protective effect is abolished by PINK1 silencing or the mitophagy inhibitor mdivi-1, confirming that Tug1 promotes pyroptosis by impairing mitochondrial quality control (181).

## 7 Challenges and considerations for clinical translation: from technical hurdles to biological complexity

### 7.1 Technical bottlenecks in exosome production, storage, and delivery for ncRNA therapeutics

A comparative summary of these intricate mechanisms, detailing the functions and targets of key ncRNAs in preclinical neuroinflammation models, is provided in **Table 4**. Notwithstanding these promising mechanistic insights, the therapeutic promise of exosome-based ncRNA delivery hinges on overcoming substantial technical challenges in production, storage, and delivery. The therapeutic promise of exosome-based ncRNA delivery hinges on overcoming substantial technical challenges in production, storage, and delivery. Exosome isolation exemplifies these hurdles, as current techniques struggle to balance efficiency and integrity. Conventional methods, such as ultracentrifugation (UC) and ultrafiltration (UF), yield low recovery rates—typically 5–25% for UC—while risking membrane damage and vesicle deformation. Emerging alternatives, including microfluidic chips, offer rapid separation in under five minutes but demand sophisticated instrumentation, limiting scalability. Immunoaffinity purification achieves exceptional purity (>90%) yet relies on costly, specific antibodies (182). To address these limitations, multi-technique integration strategies, such as sequential centrifugation combined with UF and UC, have become essential for producing high-purity, structurally intact exosomes (183). Success in these approaches requires careful optimization of six critical parameters: recovery rate, sample compatibility, molecular integrity, separation efficiency, cost-effectiveness, and operational simplicity (184). However, unlike synthetic drugs, exosome production is fraught with industrialization risks stemming from biological variability, which complicates batch-to-batch consistency. Each phase—from cell culture to harvest—profoundly influences yield and quality, necessitating stringent oversight to meet clinical standards (185).

**Table 4:** Comparative summary of ncRNA functions and targets in IS-associated neuroinflammation across preclinical models.

ncRNA	Key Target(s) / Pathway(s)	Primary Function	Expression	Model	Reference
circRNA					
CDR1as	miR-7 / α-Syn / TLR4/NF-κB	Anti-inflammatory	↓	MCAO	(96)
circ_0000831	miR-16-5p / AdipoR2 / PPARγ	Anti-inflammatory	↓	MCAO/OGD	(97)
circ-Ptpn4	miR-153-3p / Nrf2	Promotes M2 polarization	-	MCAO、OGD	(149)
lncRNA OIP5-AS1	TXNIP/​NLRP3	Inhibits pyroptosis	↓	OGD/R、MCAO/R	(176)
circFndc3b	ENO1 / Klf2 / NLRP3	Inhibits pyroptosis	↓	OGD/R、MCAO	(175)
circ_0129657	miR-194-5p / GMFB	Pro-inflammatory	↑	OGD	(98)
circZfp609	miR-145a-5p / BACH1	Pro-inflammatory	↑	MCAO/OGD	(99, 100)
circ_0007290	miR-496 / PDCD4	Pro-inflammatory	↑	AIS/OGD	(101)
circ_0000566	miR-18a-5p / ACVR2B	Pro-inflammatory	↑	OGD/R	(102)
circ_0008146	miR-709 / CX3CR1	Pro-inflammatory	↑	MCAO/LPS	(103)
circ_0000647	miR-126-5p / TRAF3	Pro-inflammatory	↑	OGD/R	(104)
circPTP4A2	STAT3 phosphorylation	Drives M1 polarization	↑	tMCAO、OGD/R	(154)
circ_0000495	miR-579-3p / TLR4/NF-κB	Drives M1 polarization	↑	OGD、MCAO	(155)
circDnajc1	miR-27a-5p / C1qc / C3/C5aR	Drives M1 polarization	↑	OGD/R、MCAO/R	(156)
lncRNA					
lncRNA SERPINB9P1	HSPA2 / TLR pathway	Anti-inflammatory	↓	OGD/R	(115)
lncRNA Maclpil	LCP1-mediated actin dynamics	Anti-inflammatory	↓	MCAO	(111)
lncRNA KLF3-AS1	miR-206 / USP22/Sirt1 or Sphk1/S1P/TRAF2/NF-κB	Anti-inflammatory	↓	MCAO/OGD/R	(112, 113)
lncRNA OIP5-AS1	miR-155-5p / IRF2BP2	Anti-inflammatory	↓	OGD/R	(114)
lncRNA SNHG16	miR-421 / XIAP	Anti-inflammatory	↓	OGD/R	(116)
lncRNA ISA1	SOCS3 / JAK2/STAT3	Promotes M2 polarization	↓	OGD/R、MCAO	(150)
lncRNA Nespas	miR-383-3p / SHP2 / NLRP3	Promotes M2 polarization	-	MCAO、OGD/R	(151)
lncRNA H19	miR-29b / C1QTNF6	Pro-inflammatory	↑	MCAO/OGD	(117)
lncRNA XIST	miR-25-3p / TRAF3	Pro-inflammatory	↑	OGD/R	(118)
lncRNA 826	Hippo pathway / YAP/TAZ	Drives M1 polarization	↑	OGD、MCAO	(157)
lncRNA NEAT1	AMPK / NF-κB	Drives M1 polarization	-	LPS	(158)
lncRNA MEG3	NLRP3/caspase-1/GSDMD	Induces pyroptosis	↑	OGD/R、MCAO/R	(179)
LOC102555978	miR-3584-5p / NLRP3	Induces pyroptosis	↑	OGD/R、MCAO/R	(180)
lncRNA Tug1	PINK1/Parkin-mediated mitophagy	Induces pyroptosis	↑	OGD/R	(181)
miRNA					
miR-149	TNF-α/IL-6 mRNAs	Anti-inflammatory	↓	MCAO	(126)
miR-30c-5p	GNAI2 / NF-κB	Anti-inflammatory	↓	OGD/R、MCAO/R	(127)
miR-124-5p	FoxO1	Anti-inflammatory	↓	MCAO/R、OGD	(128)
miR-193a-5p	UBE2V2 / PPARγ	Promotes M2 polarization	↓	MCAO、LPS	(123)
miR-369-3p	PDE4D / cAMP/PKA/AMPK	Anti-inflammatory	-	MCAO/R、OGD/R	(124)
miR-182-5p	Rac1 / NF-κB/NOX2	Anti-inflammatory	-	MCAO/R、OGD/R	(129)
miR-124-3p	TRAF6 / NF-κB	Promotes M2 polarization	↓	OGD、MCAO	(152)
miR-103-3p	TANK / NF-κB	Anti-inflammatory	↓	OGD/R、MCAO	(153)
miR-378a-5p	NLRP3-Caspase-1-GSDMD axis	Inhibits pyroptosis	-	OGD、MCAO	(177)
miR-96-5p	CASP1 / GSDMD	Inhibits pyroptosis	↓	OGD、MCAO	(178)
miR-6328	IKKβ / NF-κB	Anti-inflammatory	↑	MCAO/R、OGD/R	(125)
miR-15a/16-1	Pro-apoptotic genes	Pro-inflammatory	↑	MCAO	(130)
miR-188-5p	Lin28a	Pro-inflammatory	↑	MCAO/R、OGD/R	(134)
miR-30c-2-3p	SMAD2 / TGF-β pathway	Pro-inflammatory	↑	tMCAO	(131, 132)
miR-9-5p	Pro-inflammatory cytokines (IL-1β, TNF-α)	Pro-inflammatory	↑	LPS	(135)
miR-Novel-3	STRN / PI3K-AKT-mTOR	Pro-inflammatory (ferroptosis)	↑	MCAO、OGD	(133)
miR-210	TET2 / NF-κB	Drives M1 polarization	↑	MCAO	(159)
miR-100-5p	TLR7/NF-κB	Drives M1 polarization	↑	MCAO、OGD/R	(160)

↑ : Upregulation | ↓: Downregulation | —: Not mentioned.

Storage poses another formidable challenge, as exosomes are highly susceptible to degradation. Short-term storage at 4 °C suffices for less than 48 hours, but long-term preservation demands deep-freezing at -80 °C. Even under these conditions, exosomes suffer biofunctional decay, reduced concentration, vesicle fusion, and structural fragmentation. Prolonged storage or repeated freeze-thaw cycles exacerbate these issues, triggering aggregation that increases particle size and diminishes biological activity. Such degradation not only compromises therapeutic potential but also heightens immunogenicity risks through membrane rupture and cargo leakage (186). To mitigate these effects, protective strategies are indispensable. Storing exosomes at -80 °C in biocompatible cryoprotectants, such as 5% sucrose solution, outperforms PBS by better preserving size distribution, concentration, and membrane protein integrity. Complementing this, programmed gradient cooling at 1 °C/min suppresses ice crystal formation, reducing vesicle breakage and enhancing stability during extended storage (187).

Even with optimized production and storage, delivering ncRNA therapeutics via exosomes remains a complex endeavor. Nucleic acids face inherent vulnerabilities—nuclease degradation, poor stability, and limited transmembrane transport due to their negative charge—which impede efficient targeting and therapeutic impact (188). Achieving precise tissue- or cell-specific delivery is vital to limit off-target effects and systemic toxicity, presenting substantial challenges for delivery system design (189). Platforms like PEGylated nanoparticles improve circulation time and exploit the EPR effect (190), while focused ultrasound (FUS) enhances BBB penetration (191, 192). However, challenges such as immunogenicity persist.

Exosomes, as endogenous extracellular vesicles, offer a compelling solution, leveraging their natural nucleic acid-loading capacity, low immunogenicity, and tissue tropism. In preclinical studies of IS, intravenous injection predominates as the delivery route. Yet, natural exosomes encounter rapid clearance by the MPS, driven by plasma-derived opsonins binding to their hydrophobic surface domains. Consequently, over 90% of injected exosomes accumulate in peripheral organs, primarily the liver and spleen, leaving less than 1% to reach the ischemic brain parenchyma (193, 194). This nonspecific biodistribution undermines neuroprotective, anti-inflammatory, and pro-angiogenic effects while raising concerns about dose-dependent toxicity. Attempts to elevate brain concentrations through higher doses result in off-target accumulation in hepatic macrophages and splenic antigen-presenting cells, potentially triggering hepatotoxicity and systemic inflammation.

To overcome these delivery barriers, innovative engineering strategies have emerged. Conjugating “don’t-eat-me” signals, such as CD47 or CD55, reduces phagocytic clearance (195), while functionalizing exosomes with targeting ligands like RGD peptides enhances site-specific accumulation (196). Exploring alternative administration routes, such as intranasal delivery via the olfactory pathway, improves BBB penetration and brain enrichment. Advances in drug loading, exemplified by magnetic extrusion-derived exosome-mimetics using the ammonium sulfate gradient method, integrate enhanced payload capacity with precise targeting. Additionally, selecting specific cellular sources, such as neuron-derived exosomes bearing neural adhesion molecules, capitalizes on inherent BBB-crossing capabilities (197).These modifications collectively aim to refine delivery precision and efficacy.

Despite such progress, translating engineered exosomes into clinical practice remains hindered by persistent obstacles. Production at GMP scale incurs prohibitively high costs, compounded by suboptimal drug encapsulation efficiency and storage-related degradation. Complex pharmacokinetics—characterized by significant hepatic and splenic sequestration, low brain targeting, and a short half-life—further complicate therapeutic development. Addressing these challenges demands a comprehensive approach, where full-process GMP management plays a pivotal role. By enforcing rigorous raw material screening, terminal product quality control, strict aseptic operations, batch-to-batch consistency monitoring, and stability validation, this strategy ensures quality assurance and bridges critical logistical gaps. Ultimately, the clinical advancement of exosome-based ncRNA therapeutics hinges on systematically resolving these technical bottlenecks through integrated technological innovation and robust quality control.

### 7.2 The critical impact of biological heterogeneity: age, sex, and comorbidities

Biological heterogeneity, encompassing non-modifiable factors such as age, sex, and genetics, profoundly shapes the pathophysiology and progression of IS. These factors critically influence disease susceptibility, clinical presentation, and therapeutic outcomes (198, 199). Epidemiological evidence demonstrates that stroke incidence doubles every decade after age 55 (200). Sex-specific disparities are evident, with women facing a higher lifetime risk and mortality burden from stroke. Women account for 57.1% of global stroke deaths, and stroke ranks as the third leading cause of death in women over 55, compared to fifth in men (201–203). This disparity stems from female-specific risk factors, such as premature menopause and pregnancy complications, as well as amplified effects of shared comorbidities like hypertension and diabetes.

Preclinical studies reveal intricate interactions between age and sex in stroke outcomes. For instance, young female rodents show enhanced responses to neuroprotective treatments, such as reduced infarct volume with miR-15a/16–1 modulation. However, these sex differences diminish with age due to accumulated repair deficits (130). Interestingly, middle-aged female rodents exhibit greater cognitive vulnerability post-stroke but achieve optimal functional recovery with intervention (204)​​. These findings underscore the role of estrogen-mediated neuroprotection and the age-dependent decline in neural plasticity.

Comorbidities, particularly hypertension and diabetes, further complicate the impact of biological heterogeneity on stroke. Hypertension escalates stroke risk in a dose-dependent manner (205), while diabetes independently doubles the risk and hastens stroke onset, especially in younger individuals (205, 206). Importantly, these comorbidities interact with aging to alter therapeutic efficacy. For example, the effectiveness of FosDT siRNA diminishes in aged hypertensive models and necessitates dose adjustments in diabetic contexts. This is attributed to accelerated aging of the neurovascular unit via the NF-κB-FosDT-REST inflammatory axis (207).

Diabetes significantly worsens stroke-induced neurovascular damage through mechanisms such as metabolic dysregulation, BBB disruption, white matter injury, and heightened inflammation. These factors not only impair recovery but also reduce the efficacy of standard neurorestorative therapies, such as exosomes derived from non-diabetic mesenchymal stem cells (208). However, exosomes from diabetic rat mesenchymal stem cells (T2DM-MSC-Exo) have shown unique therapeutic benefits in diabetic stroke models. They restore BBB integrity, reduce hemorrhage, attenuate neuroinflammation, and promote white matter repair through pathways involving miR-9/ABCA1 and IGF1R (209). Additionally, diabetes exacerbates post-stroke cardiac dysfunction via impaired miR-126 signaling, a deficit that exosome therapy can address by targeting multi-organ inflammatory cascades (28). These findings highlight how metabolic dysregulation, microvascular damage, and chronic inflammation compromise recovery in patients with comorbidities (208).

A major barrier to translating stroke therapeutics from bench to bedside is the reliance on preclinical models that overlook biological heterogeneity. While most studies employ young, healthy rodents, stroke primarily affects elderly individuals with multiple comorbidities (210). This mismatch is critical, as systematic reviews indicate that less than 10% of preclinical studies test interventions in comorbid models, where therapeutic efficacy is often significantly reduced (210).

Bridging this translational gap requires the adoption of more clinically relevant preclinical models. This entails using aged animals, ensuring balanced sex representation, and incorporating validated comorbidities such as hypertension and diabetes (210–212). Moreover, given species-specific physiological differences, advanced models like non-human primates (NHPs) are crucial. For instance, pharmacokinetic studies in pig-tailed macaques show that human exosomes have a circulation half-life of approximately 40 minutes—four times longer than in rodents—and display unique immune interactions, such as rapid binding to B lymphocytes (213). These insights highlight the shortcomings of rodent models for assessing biodistribution and underscore the value of NHPs in evaluating exosome pharmacodynamics to facilitate clinical translation.

To further address the translational challenges, **Table 5** provides a systematic comparison of dysregulation patterns and functional consistency of key exosome/ncRNA regulators across preclinical and human contexts. This analysis identifies both promising concordant targets, such as circ_0000831 and miR-193a-5p, and critical discrepancies, like those involving lncRNA GAS5, which necessitate validation in human models.

**Table 5:** Comparison of key findings on exosome/ncRNA regulation of neuroinflammation in preclinical models vs. human ischemic stroke.

ncRNA	Molecular Mechanism	Model Findings	Human Disease Findings	Consistency	References
​​circ_0000831​​	Sponges miR-16-5p; activates AdipoR2/PPARγ	MCAO/OGD: ↓ expression; ↑ expression reduces apoptosis & inflammation (TNF-α, IL-1β)	IS: ↓ expression in blood	​​ Consistent	(97)
​​circ_0007290​​	Sponges miR-496; ↑ PDCD4	OGD: ↑ expression; inhibition ↓ inflammation & cell death	AIS: ↑ expression in blood	​​ Consistent	(101)
lncRNA SERPINB9P1	Binds HSPA2; ↓ TLR signaling	OGD/R: ↓ expression; ↑ expression ↑ viability & ↓ cytokines (TNF-α, IL-6)	IS: ↓ expression in blood	Consistent	(115)
miR-193a-5p	Targets UBE2V2, Stabilizes PPARγ	MCAO/LPS: ↓ expression; Overexpression ↓ infarct & cytokines (TNF-α, IL-1β)	IS: ↓ expression in blood	​​ Consistent	(123)
miR-30c-2-3p	Targets SMAD2; blocks TGF-β anti-inflammation	MCAO/OGD-R: Foam cell-derived exosomes deliver miR-30c-2-3p → ↑ inflammation	LAA-AIS: ↑ levels in plasma exosomes, positively correlated with carotid plaque instability.	Consistent	(131, 132)
miR-Novel-3	Targets STRN; ↓ PI3K-AKT-mTOR	MCAO/OGD-R: AS-exosomes deliver Novel-3 → ↑ ferroptosis & inflammation	LAA-AIS: ↑ levels in plasma exosomes, positively correlated with carotid plaque instability.	​​ Consistent	(133)
OIP5-AS1	Binds TXNIP; ↑ ubiquitin degradation	MCAO/OGD-R: ↓ expression; M2-exosomes delivery ↓ pyroptosis	IS: ↓ expression in blood; levels negatively correlate with severity	Consistent	(176)
lncRNA GAS5	Sponges miR-148b-5p; activates FCGR3	MCAO: ↑ expression; GAS5 knockdown ↓ inflammation & ↑ neuroprotection	AIS: ↓ plasma expression	Inconsistent (tissue vs. blood)	(219)

↑: Upregulation | ↓: Downregulation.

### 7.3 Optimizing exosome sources for ncRNA delivery: stem cells vs. differentiated cells

The choice of exosome source is pivotal in optimizing ncRNA delivery for treating cerebral ischemia, as different cellular origins confer distinct therapeutic advantages and limitations. Both stem cell-derived and differentiated cell-derived exosomes have demonstrated potential in promoting neural plasticity and functional recovery in stroke models, largely through their cargo of ncRNAs and bioactive lipids, which mitigate inflammation and secondary neuronal damage (214).

For instance, mesenchymal stem cell (MSC)-derived and M2 microglia-derived exosomes both deliver miR-124 but exert their effects through divergent mechanisms. MSC-derived exosomes, when engineered with rabies virus glycoprotein (RVG) for enhanced targeting, penetrate the BBB more effectively and accumulate in ischemic lesions. This promotes neurogenesis and angiogenesis while indirectly ameliorating the inflammatory microenvironment (215). In contrast, M2 microglia-derived exosomes directly modulate neuroinflammation by reducing pro-inflammatory cytokines (IL-1β, TNF-α), elevating anti-inflammatory factors (IL-10, TGF-β), and inducing microglial polarization toward the M2 phenotype. However, their therapeutic impact requires repeated administration, indicating limited persistence *in vivo* (216).

Similarly, brain endothelial cell-derived exosomes (EC-Exo) and adipose-derived stem cell exosomes (ADSC-Exo) exhibit distinct properties in delivering miR-126. EC-Exo, naturally enriched with miR-126, efficiently crosses the damaged BBB and accumulates in endothelial cells and neurons within the ischemic border zone. By enhancing vascular integrity and promoting M2 macrophage polarization, EC-Exo reverses hyperglycemia-induced miR-126 deficiency and neurovascular damage in diabetic stroke models (28). Yet, the challenge of isolating brain endothelial cells constrains large-scale production. Conversely, ADSC-Exo requires genetic modification to boost miR-126 loading and, despite weaker BBB penetration, effectively suppresses microglial activation and pro-inflammatory cytokine secretion, indirectly fostering neurogenesis and angiogenesis (27). The ease of sourcing ADSC-Exo from adipose tissue supports clinical scalability, though repeated administration is necessary to sustain efficacy, suggesting rapid clearance.

In summary, stem cell-derived exosomes, such as those from MSCs and ADSCs, offer versatility through their amenability to engineering and their capacity for multi-dimensional repair—synergistically addressing inflammation, BBB protection, and neural/vascular regeneration. This makes them well-suited for complex, multi-target conditions like stroke. Differentiated cell-derived exosomes, such as those from M2 microglia and brain endothelial cells, provide precision by directly regulating specific pathways (e.g., microglial polarization or vascular reconstruction). However, their clinical translation is hindered by production challenges, source scarcity, and pathology-dependent risks. The optimal exosome source must therefore be selected based on a careful evaluation of targeting needs, therapeutic mechanism breadth, production feasibility, and administration logistics.

## 8 Discussion

IS remains a leading global cause of death and disability, characterized by brain tissue necrosis and neuronal loss. As explored in earlier sections, ncRNAs—including circRNAs, lncRNAs, and miRNAs—orchestrate the intricate inflammatory cascade central to stroke pathology and recovery. Operating through sophisticated molecular networks, these ncRNAs modulate neuroinflammatory signaling, influencing processes such as microglial polarization, cytokine production, neuronal survival, and vascular integrity. Yet, their therapeutic application hinges on overcoming the challenge of targeted delivery to the ischemic brain.

Exosomes emerge as pivotal delivery vectors in this context. These naturally occurring nanovesicles, distinguished by low immunogenicity and inherent biocompatibility, possess a unique ability to traverse the BBB. By encapsulating and protecting their ncRNA cargo, exosomes enable precise delivery to ischemic lesions and the neurovascular unit, allowing ncRNAs to reprogram detrimental immune responses—such as shifting microglial balance—while promoting reparative pathways. This exosome-ncRNA synergy marks a transformative approach to stroke therapeutics, addressing key limitations of current strategies. It extends therapeutic windows by targeting persistent secondary injury pathways, breaches the BBB for efficient CNS delivery, and finely tunes the multifaceted neuroinflammatory response. Rather than broad suppression, this modulation recalibrates the inflammatory milieu, curbing destructive pro-inflammatory cascades while supporting beneficial reparative functions, offering a nuanced strategy to mitigate ischemia/reperfusion injury and enhance neurological restoration.

The dynamic expression patterns of ncRNAs, which are fundamental to their role in regulating post-stroke neuroinflammation, combined with their stability in readily accessible biofluids such as blood and cerebrospinal fluid, render them exceptionally promising candidates for non-invasive diagnostic and prognostic biomarkers in IS (217, 218) (**Table 6**). Specific alterations in the levels of these regulatory molecules often correlate with the onset, severity, progression, and subtype of stroke, reflecting the underlying inflammatory state and tissue injury.

**Table 6:** ncRNA Biomarkers in Patient Biofluids: Diagnostic and Prognostic Potential for Ischemic Stroke-Associated Neuroinflammation.

ncRNA	Clinical Sample (Cohort Size)	Δ Expression (p-value)	Diagnostic Performance (AUC)	Reference
lncRNA MALAT1	Plasma: AIS vs. HCs (n=30)	↑ (p < 0.05)	0.915	(219)
lncRNA GAS5	Plasma: AIS vs. HCs (n=30)	↓ (p < 0.01)	0.893	(219)
miR-30a-5p	Plasma: HIS vs. HCs (n=15)	↑ (p < 0.05)	0.826	(220)
miR-21-5p	Plasma: SIS/RIS vs. HCs (n=15)	• SIS: ↑ (p < 0.05) • RIS: ↑ (p < 0.01)	• SIS: 0.714 • RIS: 0.734	(220)
miR-574-5p	Plasma: IS vs. HCs (n=103 vs. 87)	↓ (p < 0.0001)	0.866	(221)
miR-28-5p	Plasma: IS vs. HCs (n=216 vs. 102)	↑ (p < 0.0001)	0.938	(222)
lncRNA PSMB8-AS1	Plasma: IS vs. EH (n=280 vs. 260)	↑ (p < 0.001)	0.954	(223)

↑: Upregulation | ↓: Downregulation

Among promising ncRNA biomarker candidates, lncRNAs MALAT1 and GAS5 highlight both potential and complexity. In mouse MCAO models, both are upregulated in brain tissue, with MALAT1 knockout reducing neurological deficits, inflammation (e.g., IL-1β, TNF-α), and enhancing neuronal survival, indicating it worsens injury via inflammation, while GAS5 knockout curbs neuroinflammation and promotes neuroprotection (219). Yet, in AIS patient serum, MALAT1 rises while GAS5 falls, revealing a stark model-clinical divergence. This discrepancy, tied to tissue-specific regulation and species differences, complicates biomarker translation, though their opposing serum shifts, linked to disease and inflammation, affirm their diagnostic potential for AIS.

Nevertheless, their significant and opposing changes in patient serum, correlating with disease status and inflammation, underscore their strong potential as inflammation-linked diagnostic indicators for AIS Demonstrating temporal diagnostic utility, miR-30a-5p is specifically upregulated in the hyperacute stage (HIS) with excellent diagnostic efficacy (AUC=0.826), positioning it as a potential HIS-specific biomarker. Conversely, miR-21-5p shows sustained elevation during the subacute (SIS) and recovery (RIS) stages, likely linked to neuroprotective responses. Critically, combining these two miRNAs effectively distinguishes between HIS, SIS, and RIS stages, offering a high-value, non-invasive tool for stage-specific diagnosis with significant clinical translation prospects (220). Numerous other ncRNAs show significant diagnostic and/or prognostic value. For instance, serum miR-574-5p levels are significantly lower in IS patients, correlating negatively with neurological deficit severity (NIHSS) and serving as an independent risk factor for poor prognosis (221); serum HDL-C-associated miR-28-5p levels are elevated in AIS patients, negatively correlating with HDL-C (r = -0.848), demonstrating high diagnostic efficacy (AUC=0.938) and correlation with stroke severity (r=0.777) (222); and serum lncRNA PSMB8-AS1 is significantly elevated in IS versus hypertension alone (P < 0.001), showing high diagnostic accuracy (AUC=0.954), correlating with neurological deficit severity, and strongly predicting poor prognosis (mRS>2; AUC=0.888, sensitivity 97.8%) and increased 12-month recurrence risk (223).

While neuroimaging (CT/MRI) remains the diagnostic cornerstone for IS ncRNA detection emerges as a highly promising complementary approach. It offers a pathway towards earlier detection (particularly valuable in the hyperacute phase where imaging may be equivocal), enhanced sensitivity, non-invasiveness, and personalized prognosis, potentially addressing limitations like accessibility and cost. However, challenges remain: ncRNA expression can be influenced by comorbid conditions or other neurological injuries, and the precise assessment of disease progression and prognosis using specific further large-scale validation and refinement.

### 8.1 Limitations

While providing a systematic synthesis, this review has the following limitations: (i) It focuses primarily on neuroinflammation, not encompassing all stroke pathologies; (ii) Its conclusions rely solely on English-language studies from PubMed, potentially omitting relevant unpublished data or non-English findings, and it did not perform a meta-analysis, limiting statistical power and formal assessment of heterogeneity and publication bias; (iii) The field’s rapid evolution means new discoveries may supersede included evidence; (iv) Interpretation is constrained by inconsistent reporting of statistical parameters (e.g., power analysis, multiple testing corrections, sample size justification) across studies, variability in study quality, potential publication bias (underrepresentation of negative/null results), and low external/internal validity across diverse preclinical models; (v) Clinical biomarker data, though promising, require extensive validation.

## 9 Conclusions

Exosomes and ncRNAs constitute a groundbreaking frontier in understanding and managing post-IS neuroinflammation. Their capacity to precisely regulate inflammatory networks, penetrate the BBB, and serve as biomarkers offers unparalleled opportunities to address the shortcomings of existing stroke therapies. Despite persistent challenges in production, delivery, biological heterogeneity, and clinical validation, the mechanistic insights presented here lay a robust foundation. Through innovative engineering, rigorous preclinical testing in clinically relevant models, and strategic clinical translation, the full potential of exosome-ncRNA strategies can be realized, ultimately enhancing functional recovery and quality of life for stroke survivors.
